# Robust Wavelength Selection Using Filter-Wrapper Method and Input Scaling on Near Infrared Spectral Data †

**DOI:** 10.3390/s20175001

**Published:** 2020-09-03

**Authors:** Divo Dharma Silalahi, Habshah Midi, Jayanthi Arasan, Mohd Shafie Mustafa, Jean-Pierre Caliman

**Affiliations:** 1 SMART Research Institute, PT. SMART TBK, Pekanbaru 28289, Riau, Indonesia; divosilalahi@yahoo.co.id (D.D.S.); caliman@indo.net.id (J.-P.C.); 2 Institute for Mathematical Research, Universiti Putra Malaysia (UPM), Serdang 43400, Selangor, Malaysia; jayanthi@upm.edu.my (J.A.); mshafie@upm.edu.my (M.S.M.); 3 Department of Mathematics, Faculty of Science, Universiti Putra Malaysia (UPM), Serdang 43400, Selangor, Malaysia

**Keywords:** near infrared spectral data, robust statistics, partial least squares, scaling, variable selection, variable importance in projection, uninformative variable eliminations

## Abstract

The extraction of relevant wavelengths from a large dataset of Near Infrared Spectroscopy (NIRS) is a significant challenge in vibrational spectroscopy research. Nonetheless, this process allows the improvement in the chemical interpretability by emphasizing the chemical entities related to the chemical parameters of samples. With the complexity in the dataset, it may be possible that irrelevant wavelengths are still included in the multivariate calibration. This yields the computational process to become unnecessary complex and decreases the accuracy and robustness of the model. In multivariate analysis, Partial Least Square Regression (PLSR) is a method commonly used to build a predictive model from NIR spectral data. However, in the PLSR method and common commercial chemometrics software, there is no standard wavelength selection procedure applied to screen the irrelevant wavelengths. In this study, a new robust wavelength selection procedure called the modified VIP-MCUVE (mod-VIP-MCUVE) using Filter-Wrapper method and input scaling strategy is introduced. The proposed method combines the modified Variable Importance in Projection (VIP) and modified Monte Carlo Uninformative Variable Elimination (MCUVE) to calculate the scale matrix of the input variable. The modified VIP uses the orthogonal components of Partial Least Square (PLS) in investigating the informative variable in the model by applying the amount of variation both in X and y{SSX,SSY}, simultaneously. The modified MCUVE uses a robust reliability coefficient and a robust tolerance interval in the selection procedure. To evaluate the superiority of the proposed method, the classical VIP, MCUVE, and autoscaling procedure in classical PLSR were also included in the evaluation. Using artificial data with Monte Carlo simulation and NIR spectral data of oil palm (*Elaeis guineensis* Jacq.) fruit mesocarp, the study shows that the proposed method offers advantages to improve model interpretability, to be computationally extensive, and to produce better model accuracy.

## 1. Introduction

The selection of relevant wavelengths in chemometrics analysis in Near Infrared (NIR) spectral data is crucial to prevent the number of relevant variables to be removed in the analysis. Partial Least Square Regression (PLSR), a conventional method used in chemometrics, has no standard procedure. There are related studies of its application in oil and fat assessment in oil palm (*Elaeis guineensis* Jacq.). However, many researchers are still using the band partition experiments (see [1,2,3]) by manually segmenting the wavelengths into several bands: visible (400–700 nm), NIR (701–1100 nm), Shortwave Infrared 1 (SWIR1: 1101–1351 nm), SWIR2 (1400–1800 nm), and SWIR3 (2000–2500 nm). The selection procedure is commonly done based on trial and error [1] through the improvement in model accuracy attained. This observation is inefficient and requires advanced experience. Moreover, an in-depth analysis needs to be carried out to understand the NIR spectra signature based on its chemical information. A wavelength selection method is therefore required to assess the contribution of each wavelength. This selection will reduce the number of variables used in the model. In the interpretation, the selection method may give a better understanding of the underlying process of the sample studied.

In the review papers (see [4,5,6,7]), several recommended wavelength selection methods have been discussed. The researchers have highlighted the limitations and properties of each method presented. No one has suggested which method is better than the other. A convenient approach is to make a comparison between the methods and examine their superiority using the experimental simulation and real given problems. There are three main categories of variable selection methods: filter, wrapper, and embedded. The main differences between these categories are based on their processing steps. In the filter methods, relevant variables are ranked and selected according to the threshold on the relevancy index calculated from the fitted model [8,9]. The filter methods are considered fast and straightforward because no learning algorithm is required in the computational process. However, the filter methods do not take into account biasness in the learning model and neglect any conditional dependence (or independence) that might probably exist [10]. In the wrapper methods, the supervised learning approach adopts the search algorithm iteratively with either the deterministic or randomization [5]. The wrapper methods are known to be costly because in the evaluation criterion, a predefined learning algorithm and cross-validation procedure is performed [5]. In the embedded methods, the advantages of both the filter and wrapper methods are seized [7]. The embedded methods evaluate the quality of selected relevant variables during the model building without performing an evaluation process on the learning model [11]. This embedded strategy has motivated the current study to integrate the combination steps in the filter and wrapper methods with the objective to improve the performance of the PLSR model.

There are several methods associated with both the filter and the wrapper methods. In the filter methods, some selection procedures have been reported, such as the Stepwise Regression Coefficients [12], Loading Weights [13], Correlation Coefficient [14], and Variable Importance in Projection (VIP) [15]. Among these procedures, the VIP has earned considerable attention because of its stability and consistency to select the relevant wavelengths and its relatively low computational cost. Using different dataset matrix as simulation, the VIP outperforms the other selection methods (see [6,16]). In the classical VIP [17], the score is calculated by using the weight combination of the overall component variables of the squared PLSR weight. However, the score highlights the variation only in the predictive components without including the orthogonal components. An upgrade of the VIP score based on the Orthogonal Projections to Latent Structures (OPLS) [18] was then introduced. The OLPS-VIP score systematically includes and differentiates the variation both in the predictive and orthogonal components. In some studies (see [18,19]), the OPLS-VIP has shown its superiority to the classical VIP. In the wrapper methods, some selection methods have also been discussed, such as the Genetic Algorithm (GA) [20], Uninformative Variable Elimination (UVE) [21], Iterative Predictor Weighting [22], and Backward Variable Elimination [23]. Among these methods, the UVE method is the most consistent method to improve the PLSR performance [21,24].

Nonetheless, the leave-one-out validation procedure is still applied in the classical UVE. This procedure leads to overfitting and is time-consuming while acquiring the stability values for a large dataset. As an improvement, the Monte Carlo Uninformative Variable Elimination (MCUVE) method was proposed by Cai [25]. The MCUVE adopts the principle of Monte Carlo to evaluate the stability of the corresponding coefficients. However, the reliability coefficient and the cut-off criterion performed in the method to date is not robust enough. Therefore, this has motivated the current study to propose a slight improvement to make the MCUVE procedure more robust.

In practice, it is difficult to eliminate all the irrelevant wavelengths in spectra processing. A smaller number of wavelengths (as predictor variables) used in the model calibration will result in overfitting or underfitting. To overcome this, a new robust procedure to highlight the relevant wavelengths and to downgrade the influence of the irrelevant wavelengths in the PLSR model is needed. It has been investigated that the scaling method in the PLS model is also essential to improve the convergence speed of the algorithm [26,27]. In addition, the auto-scaling method using the mean centering and standard deviation, is a common scaling procedure in the data pre-processing step. Hence, another scaling strategy should be considered in the improvement.

The main objectives of this study are (1) to establish a new procedure for wavelengths selection called the modified VIP-MCUVE (mod-VIP-MCUVE) with input scaling strategy in the PLSR model; (2) to evaluate the performance of the proposed method with the standard auto-scaling procedure in the PLSR and the input scaling strategy using the classical VIP and MCUVE methods; (3) to apply the proposed method on the artificial data and NIR spectra of oil palm fruit mesocarp (fresh and dried ground).

## 2. Materials and Methods

### 2.1. Partial Least Square Regression

Partial Least Square Regression (PLSR) was firstly initiated by Wold [28] as the generalized statistical method and standard method used in the spectroscopy analysis. Let us define a multiple regression model that relates several m predictors X to a response variable y. In matrix form this can be written as

(1) y=X b+e

where y is an n×1 vector of the response variable, X is n×m matrix of predictors, b is a m×1 vector of unknown parameters, and e is a n×1 vector of random errors. The solution for the estimator b using the least-squares method is given as

(2) $$\hat{b}={\left(X^{T}X\right)}^{-1}X^{T}y$$

Here the data set problem is in condition with a large number of m predictors. Hence, there will be an infinite number of solutions for estimating b as $X^{T}X$ is singular, which does not meet the usual trivial theorem on rank in the regression. In this case, it is necessary to extract the new latent variables by maximizing a covariance criterion between predictor X and response y that link the central values of these two sets [29].

Initialize a starting n×1 score vector of u from any single y as in Equation (1), there exists an outer relation for predictor X as

(3) $$X=VP^{T}+E$$

where P${\left\{p_{g}=\left(X^{T}\;v_{g}\right)/\left(v_{g}{}^{T}v_{g}\right)\right\}}_{g=1}^{l}$ is the matrix m×l consists of loading vector m×1, $v_{g}$${\left\{v_{g}=\left(Xw_{j}\right)/\left(w_{j}{}^{T}w_{j}\right)\right\}}_{g=1}^{l}$ is the n×1 column vector of scores $x_{j}$ in X involves $w_{j}$${\left\{w_{j}=\left(X^{T}u\right)/\left(u^{T}u\right)\right\}}_{j=1}^{m}$ as m×1 vector of weight for X, V is a n×l matrix of n×1 vector $v_{g}$, and E is a n×m matrix of residual in outer relation for predictor X. Following these, the outer relation for the response y also can be defined as

(4) $$y=u\;q^{T}+f$$

where q is the loading l×1 vector ${\left\{q_{g}=\left(y^{T}\;v_{g}\right)/\left(v_{g}{}^{T}v_{g}\right)\right\}}_{g=1}^{l}$ and f is a n×1 vector of residual in y. u${\left\{u=b_{g}\;v_{g}\right\}}_{g=1}^{l}$ is also called as linear inner relation between X and y block score, which simply can be written as

(5) $$u=V\;b_{inner}+g$$

where $b_{inner}$${\left\{b_{g}=u_{}^{T}\;v_{g}/\left(v_{g}^{T}v_{g}^{}\right)\right\}}_{g=1}^{l}$ is a l×1 vector of regression coefficient as Least Square solution on the decomposition of vector u, and g is a n×1 vector of residual in the inner relation. Applying the normalization in P, W, and q as the process to improve the inner relation, the mixed relation of PLSR model by integrating Equations (4) and (5) results as

(6) $$\begin{array}{l} y=u\;q^{T}+f\\ y=V\;b_{inner}\;q^{T}+g\;q^{T}+f\\ y=V\;b_{inner}\;q^{T}+\overset{⌣}{f}\\ y=V\;a^{T}+\overset{⌣}{f} \end{array}$$

where a$\left\{a^{T}=b_{inner}\;q^{T}\right\}$ is the l×1 vector coefficient and $\overset{⌣}{f}$$\left\{\overset{⌣}{f}=g\;q^{T}+f\right\}$ denotes n×1 vector of residual in the mixed relation. Equation (6) holds $a=V^{T}y$, and without loss of generality $X=VP^{T}$ as in Equation (3), the formulation in Equation (6) can be reconstructed by multiplying the two sides with weight matrix of W which is

(7) $$y=X\;W^{*}\;a+\overset{⌣}{f}\;y=X\;W\;{(P^{T}W)}^{-1}\;a+\overset{⌣}{f}$$

with $V=X\;W^{*}$ and $W^{*}=W(P^{T}W{)}^{-1}$.

Let us define $b_{PLSR}=W\;{(P^{T}W)}^{-1}a$ as m×1 vector coefficient of mixed relation in the PLSR, then Equation (7) is equivalent to

(8) $$y=X\;b_{PLSR}+\overset{⌣}{f}$$

where $\overset{⌣}{f}$ has to be minimized. Applying the relation in Equations (3) and (4), so that $W=X^{T}u$, $P=X^{T}V{\left(V^{T}V\right)}^{-1}$. Therefore, the estimator for the parameter $b_{PLSR}$ can be calculated as

(9) $${\hat{b}}_{PLSR}=X^{T}u{(V^{T}X\;X^{T}u)}^{-1}\;V^{T}y,\;{\hat{b}}_{PLSR}\in {ℜ}^{m\;\times 1}$$

${\hat{b}}_{PLSR}$ denotes the m dimensional vector of regression coefficient in the PLSR model.

### 2.2. Variable Selection Methods

#### 2.2.1. Variable Importance in Projection

In the classical VIP [17], the VIP score measures the contribution of each j th wavelength in the multivariate models based on the projection to the PLS components. The method becomes popular because of its simple procedure and less computational complexity [16]. The VIP score is formulized through the normalized loading weights $v_{g}$$\left\{v_{g}=w_{g}/\left\|w_{g}\right\|\right\}$ and the explained sum of squares for the predictive component y. Mathematically, the VIP score for each j th wavelength in the PLS model with l components can be calculated as

$${\mathrm{VIP}}_{j}^{2}=\frac{m\;\sum_{g=1}^{l}\left(v_{g}^{2}\;{\mathrm{SSY}}_{comp;\;g}\right)}{\sum_{g=1}^{l}{\mathrm{SSY}}_{comp;\;g}}$$

where m is the number of predictors, ${\mathrm{SSY}}_{comp;\;\;g}$ is the variance of y explained by the gth PLS component, and $\sum_{g=1}^{l}{\mathrm{SSY}}_{comp;\;g}$ is the total variance summarized by the PLS model over l components. The criteria are the j th wavelength with VIP score > 1 is considered as the most relevant, while the VIP score < 0.5 is considered as irrelevant wavelength.

#### 2.2.2. Uninformative Variable Elimination

The classical UVE method [21] uses the leave-one-out jackknife method and artificial random noise variables denoted as n×m matrix N to compute the statistic parameters. The reliability of each wavelength through variable selection criterion then is calculated based on the PLSR coefficient $b_{PLSR}=\left[b_{1},b_{2},\ldots ,b_{m}\right]$. However, when handling a large dataset, this procedure becomes costly [25,30]. As a solution, the Monte Carlo method, which is based on random selection and probability statistics, is applied in the UVE and so-called as MCUVE [25]. In the MCUVE, some specific number of a subsample $N_{t}$ from the training set are randomly selected to build the r PLS sub-model. Then, it produces the number of set of PLSR coefficient ${\hat{b}}_{PLSR}$ as r×m matrix. The reliability $c_{j}$ is computed based on the fraction between the mean and standard deviation of m×1 column vector of PLSR coefficient $b_{i*j}$ in each jth wavelength from the $i^{*}$ vectors of coefficients. The highest $c_{j}$ represents the most reliable wavelengths; otherwise, the class is as a less reliable wavelength.

(10) cj=b¯js(bj)b¯j=∑i∗=1rbi∗jrs(bj)=(∑i∗=1r(bi∗j−b¯j)2r−1)12

The cut-off threshold criterion in MCUVE is defined through the maximum absolute value of the reliability $c_{artif}$ of the artificial random noise variables matrix N. The wavelength with $c_{j}$ less than the artificial random noise $c_{artif}$ is removed from the PLSR model.

(11) $$\left|\left(c_{j}\right)\right|<\left|\max\;(c_{artif})\right|$$

## 3. Input Scaling of Filter-Wrapper Method

Auto-scaling is a common input scale method used to standardize a dataset in the modeling process of PLSR. This auto-scaling transforms each numeric in the input variable into the same variance through its mean and standard deviation [31,32]. However, this method is observed disadvantageous when the original input variables are measured on the same scale. Moreover, it removes the interpretability related to the loadings [33]. Taking a particular concern in the intensity power of Near Infrared region, auto-scaling fails to keep useful interpretive information about the wavelength contribution since the low-intensity regions are enhanced to the same magnitude as like in the high intensity [34]. The scaling method is very crucial to correct for wavelengths-dependent scattering effects in the NIR spectra dataset [35,36]. To overcome this, a new alternative input scaling method based on the Filter and Wrapper methods is proposed. This method is then simply denoted as mod-VIP-MCUVE. Besides correcting the wavelengths-dependent scattering effects and preserving the chemical interpretive information in each wavelength, the proposed method will eliminate the influence of irrelevant wavelengths during the modeling process. In general, the three main computational steps of the mod-VIP-MCUVE method are the following.

- Step 1: Calculate the OPLS-VIP [19] score as initial input variable scaling matrix.
- Step 2: Run the modified MCUVE procedure using the scaled input matrix of OPLS-VIP to get the reliability scores.
- Step 3: Re-scale the input matrix using the reliability scores as final scaled input matrix in the PLSR model.

In the OPLS-VIP [19], the VIP score is measured not only based on the projections to the PLS components but also included the orthogonal components. This score considers variations both in the predictor variable X(SSX) and the response variable y(SSY). Here, the fourth variant of four versions of OPLS-VIP is preferred due to its interpretative information ability. The OPLS-VIP score uses the combinations {SSX,SSY} in the weighting parameters and normalized loadings $v_{g}$. The total OPLS-VIP score then is used as the final VIP score as it is calculated based on the ${\mathrm{VIP}}_{pred}$ (predictive components) and ${\mathrm{VIP}}_{ortho}$ (orthogonal components).

Let us redefine g as the predictive component and $g_{o}$ as the orthogonal component, then l stands for the total number of predictive components, and $l_{o}$ stands for the total number of orthogonal components with m and $m_{o}$ are the total number of variables used in the predictive and orthogonal components, respectively. The formulation for OPLS-VIP score both in predictive and orthogonal can be written as

(12) $${\mathrm{VIP}}_{pred}=\sqrt{\frac{m}{2}\left(\frac{\sum_{g=1}^{l}\left(v_{{}_{g}}^{2}\;{\mathrm{SSX}}_{comp;\;g}\right)}{{\mathrm{SSX}}_{cum}}+\frac{\sum_{g=1}^{l}\left(v_{{}_{g}}^{2}\;{\mathrm{SSY}}_{comp;\;g}\right)}{{\mathrm{SSY}}_{cum}}\right)}$$

(13) $${\mathrm{VIP}}_{ortho}=\sqrt{\frac{m_{o}}{2}\;\left(\frac{\sum_{g_{o}=1}^{l_{o}}\left(v_{o}{}_{{}_{g_{o}}}^{2}\;{\mathrm{SSX}}_{comp;\;g_{o}}\right)}{{\mathrm{SSX}}_{cum}}+\frac{\sum_{g_{o}=1}^{l_{o}}\left(v_{o}{}_{{}_{g_{o}}}^{2}\;{\mathrm{SSY}}_{comp;\;g_{o}}\right)}{{\mathrm{SSY}}_{cum}}\right)}$$

where, as in Equations (12) and (13), the sum of square (SS) both in variable y and variable X has subscript comp; g and $comp;\;g_{0}$. The subscript comp; g refers to the explained SS of gth component in the predictive, while the subscript $comp;\;g_{0}$ refers to the explained SS of $g_{o}\mathrm{th}$ component in the orthogonal. The SS with subscript cum refers to the cumulative explained SS overall components in the model. The total of OPLS-VIP score (denotes as VIP-total) is then formulized as

(14) $${\mathrm{VIP}}_{total}=\sqrt{\frac{M}{2}\;\left(\begin{array}{l} \frac{\sum_{g_{o}=1}^{l_{o}}\left(v_{o}{}_{{}_{g_{o}}}^{2}\;{\mathrm{SSX}}_{comp;\;g_{o}}\right)}{{\mathrm{SSX}}_{cum}}+\frac{\sum_{g=1}^{l}\left(v_{{}_{g}}^{2}\;{\mathrm{SSX}}_{comp;\;g}\right)}{{\mathrm{SSX}}_{cum}}+\\ \frac{\sum_{g_{o}=1}^{l_{o}}\left(v_{o}{}_{{}_{g_{o}}}^{2}\;{\mathrm{SSY}}_{comp;\;g_{o}}\right)}{{\mathrm{SSY}}_{cum}}+\frac{\sum_{g=1}^{l}\left(v_{{}_{g}}^{2}\;{\mathrm{SSY}}_{comp;\;g}\right)}{{\mathrm{SSY}}_{cum}} \end{array}\right)}$$

where M is the sum of variables used both in the predictive and orthogonal components

$$\left\{m=M/\left(\frac{{\mathrm{SSX}}_{cum;\;g}}{{\mathrm{SSX}}_{cum}}+\frac{{\mathrm{SSY}}_{cum;\;g}}{{\mathrm{SSY}}_{cum}}\right)\right\};\;\left\{m_{0}=M/\left(\frac{{\mathrm{SSX}}_{cum;\;g_{o}}}{{\mathrm{SSX}}_{cum}}+\frac{{\mathrm{SSY}}_{cum;\;g_{o}}}{{\mathrm{SSY}}_{cum}}\right)\right\}$$

The above total OPLS-VIP score is used to scale the original wavelength variables as the new input matrix. Let us define $\tilde{X}$ as the scaled input variable that is calculated by using the OPLS-VIP score on predictor variable X which are initially not scaled. Mathematically it can be written as

(15) $$\tilde{X}=X\;\Omega \;\Omega =\mathrm{diag}\left(\lambda_{1},\lambda_{2},\ldots ,\lambda_{m}\right)$$

where Ω∈ℜ is said to be a diagonal weight matrix with size m×m. The element $\lambda_{j}$ in the diagonal matrix Ω is a non-negative variable scaling factor for the jth input wavelength. The new scaled $\tilde{X}$ is used as a new input matrix in the modified MCUVE.

In the modified MCUVE, the drawback of the classical cut-off threshold criterion in Equation (11) has been discussed by Centner (see [21]). As an alternative, a new modified robust cut-off criterion based on a one-sided tolerance interval from Natrella [37] is proposed with a better stable elimination on the irrelevant wavelengths. The cut-off value is calculated using the robust location and scale of the reliability coefficients obtained from the added artificial uninformative random variable. In addition, it includes the value of k factor as a function of the γ desired proportions, α as a level of error, and r number of repetition used in MC random subsample selection. Using the $c_{artif}$ in Equation (11), then the new proposed cut-off criterion can be defined as

(16) $$\mathrm{cut}\text{-}\mathrm{off}\;\mathrm{value}=\mathrm{median}\;({\left(c_{j}\right)}_{artif})+k\;(\mathrm{MAD}\;{\left(c_{j}\right)}_{artif})$$

where k can be calculated as

$$k=\frac{z_{\gamma }+\sqrt{z_{\gamma }^{2}-a\;b}}{a}$$

With constant parameters $\left\{a=1-\frac{z_{\alpha }^{2}}{2(r-1)}\right\}$ and $\left\{b=z_{\gamma }^{2}-\frac{z_{\alpha }^{2}}{r}\right\}$. This new cut-off criterion benefits from classifying the most ($c_{j}>\mathrm{cut}-\mathrm{off}\;\mathrm{value}$) and less relevant variable for further interpretation. Applying the reliability $c_{j}$ of modified MCUVE as the element $\lambda_{j}$ in Ω, the new scaled input variable ${\tilde{X}}^{*}$ for the PLSR model is then updated.

## 4. Monte Carlo Simulation Study

A simulation study was carried out to evaluate the performance of our proposed method and to compare its performance with some existing methods discussed in this study. Following the simulation study of Kim [26], the artificial dataset was generated randomly using the Uniform distribution (0,1) and included the added noise that follows the normal distribution. This dataset was applied in the linear combination equation with different scenarios. Five sample sizes (n= 40, 60, 150, 400, and 600), three levels of number of predictor variables (m=41, 101, and 201), and five levels of number of important variables (*IV* = 0.10, 0.20, 0.40, 0.60, and 0.80) were considered. The 100 (IV) % of predictor variables were selected as important variables, and the remaining 100 (1−IV) % were considered as less important. The formulation of this simulation can be defined as follows

(17) $$\begin{array}{l} m=mo+me\\ c_{j^{o}}\sim U\;\left(1,\;10\right)\;\;\;\;\;\;\;\;\left(j^{o}=1,\;2,\;\ldots ,\;mo\right)\\ ce_{j^{e}}\sim U\;\left(5,\;20\right)\;\;\;\;\;\left(j^{e}=1,\;2,\;\ldots ,\;me\right)\\ e_{j}\sim N\;\left(0,1\right)\;\;\;\;\;\;\left(j^{}=0,\;1,\;2,\ldots ,\;m\right)\\ b\sim U\left(0,7\right)\\ X=\left\{c_{j^{o}},ce_{j^{e}}\right\}+e_{j}\;\;\;\left(j=1,\;2,\ldots ,\;m;j^{o}=1,\;2,\;\ldots ,\;mo;j^{e}=1,\;2,\;\ldots ,\;me\right)\\ iv=\left\{\;iv_{1},iv_{2},\ldots ,\;iv_{100(IV)\%*m}\right\}\\ y=Xb+e_{0}\;\;\;\;\left(i=1,\;2,\ldots ,\;n;j=iv_{1},iv_{2},\ldots ,\;iv_{100(IV)\%*m}\right) \end{array}$$

where m is the total number of predictors, mo is the number of observable variables and the me{me=(m−100 (IV) %∗m)/2} is the number of artificial noise variable. These artificial variables are classified as less important variables in the dataset. In Equation (17), the $c_{j^{o}}$, $ce_{j^{e}}$, and $e_{j}$ are independent of each other while X and y are illustrated as observable variable. The $c_{j^{o}}$ follows the Uniform distribution (1,10) with size *n*. The artificial noise variables $ce_{j^{e}}$ are added to the predictor and follow the Uniform distribution (5,20) with size *n*. This $ce_{j^{e}}$ is classified as a less important variable. The $e_{j}$ follows the standard normal distribution with size *n* and b represents a vector coefficient for selected important variables which follows the Uniform distribution (0,7) with size *m*. The iv as the set of selected important variables in mo and $e_{0}$ is added error in the linear combination of y. In the PLSR model, the number of PLS components is a principal indicator in the modeling since it is always viewed to be subjective.

**Figure 1:**
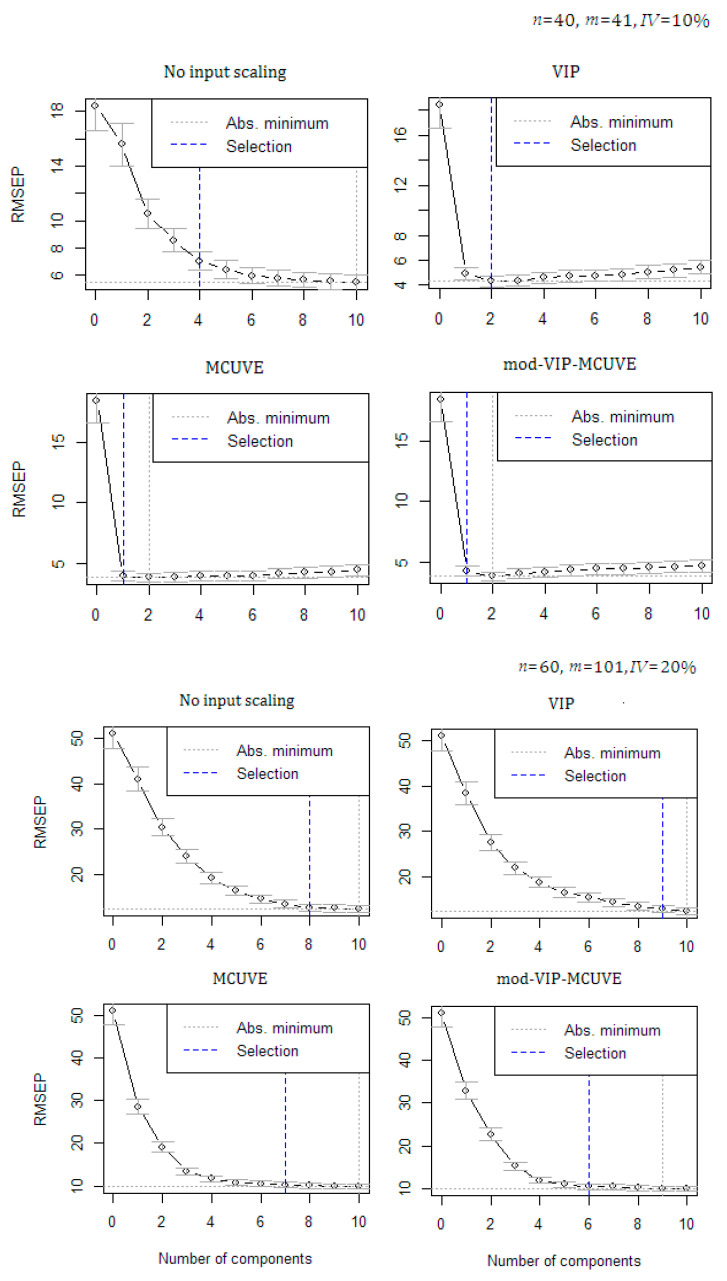

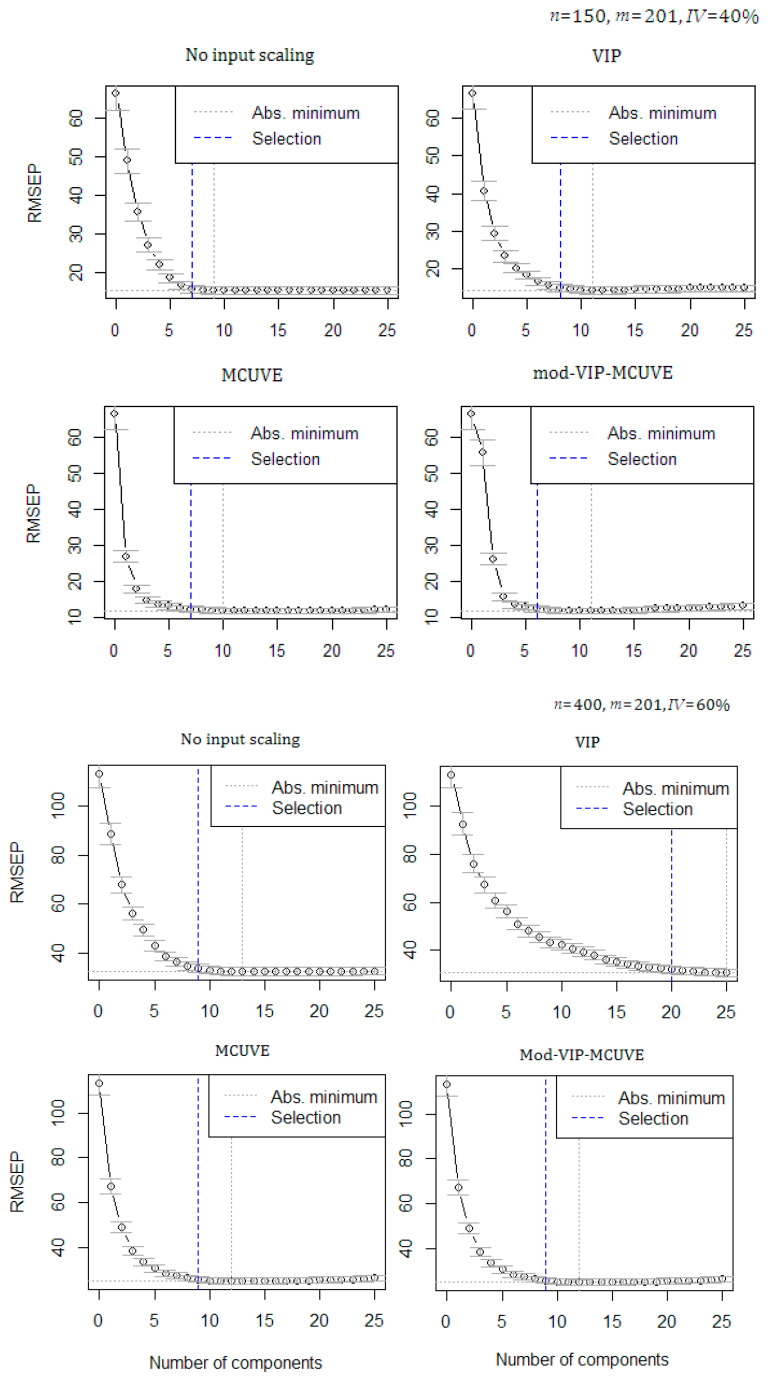
Global minimum cross-validation for the optimum number of PLS components on different dataset scenarios.

In Figure 1, a re-sampling procedure called cross-validation, showing the lowest Root Mean Square Error of Prediction (RMSEP) is used to select the optimum number of PLS components. ‘Selection’ means the selected optimum number of PLS components suggested by cross-validation techniques (highlighted with the blue dashed line). While, the ‘Abs.minimum’ refers to the lowest RMSEP (highlighted with the gray dashed line). As the number of PLS components used in the PLSR model increases, the mean of RMSEP also decreases. The optimum number of PLS components depends on how well the specific number of original variables have contribution to the model. In the experiment using different levels of n, m, and IV, the proposed mod-VIP-MCUVE requires a smaller amount of PLS components to fit minimum RMSEP than the other methods (classical PLSR method with no input scaling applied, VIP and MCUVE). The results are consistent and satisfying. The MCUVE input scaling method is comparable to the proposed method, but it still produces higher RMSEP values. When accommodating fewer variables used as predictor, a faster computational speed will be attained. Based on the global minimum cross-validation, the proposed mod-VIP-MCUVE has succeeded in reducing the RMSEP and improving the accuracy of the PLSR model.

Several statistical measures as evaluation indices are used to assess the goodness of the methods: Root Mean Square Error (RMSE), Coefficient of Determination (R^2^), Ratio of Performance to Deviation (RPD), and Standard Error (SE). The RMSE indicates the absolute measure of fit, R^2^ measures the proportion of variation in the data explained by the model, RPD assess the reliability of the goodness of fit for model, and SE measures of the uncertainty in the NIRS prediction. In this section, the Monte Carlo simulation was run 10,000 repeated times, and the results are based on the average of statistical measures (see Table 1). Some scenarios using different treatments are applied to evaluate the PLSR model. According to the results, comparing the RMSE, R^2^, and SE values in all scenarios, the proposed mod-VIP-MCUVE produced better accuracy than the other methods. The reliability of proposed method based on its RPD is still outperformed. Downgrading the irrelevant variables in the fitting process, the performance of the proposed method is comparable to the classical PLSR method with full variables involved. This shows that the proposed mod-VIP-MCUVE with fewer numbers of variables is more efficient than the traditional PLSR model since it could obtain a similar accuracy.

**Figure 2:**
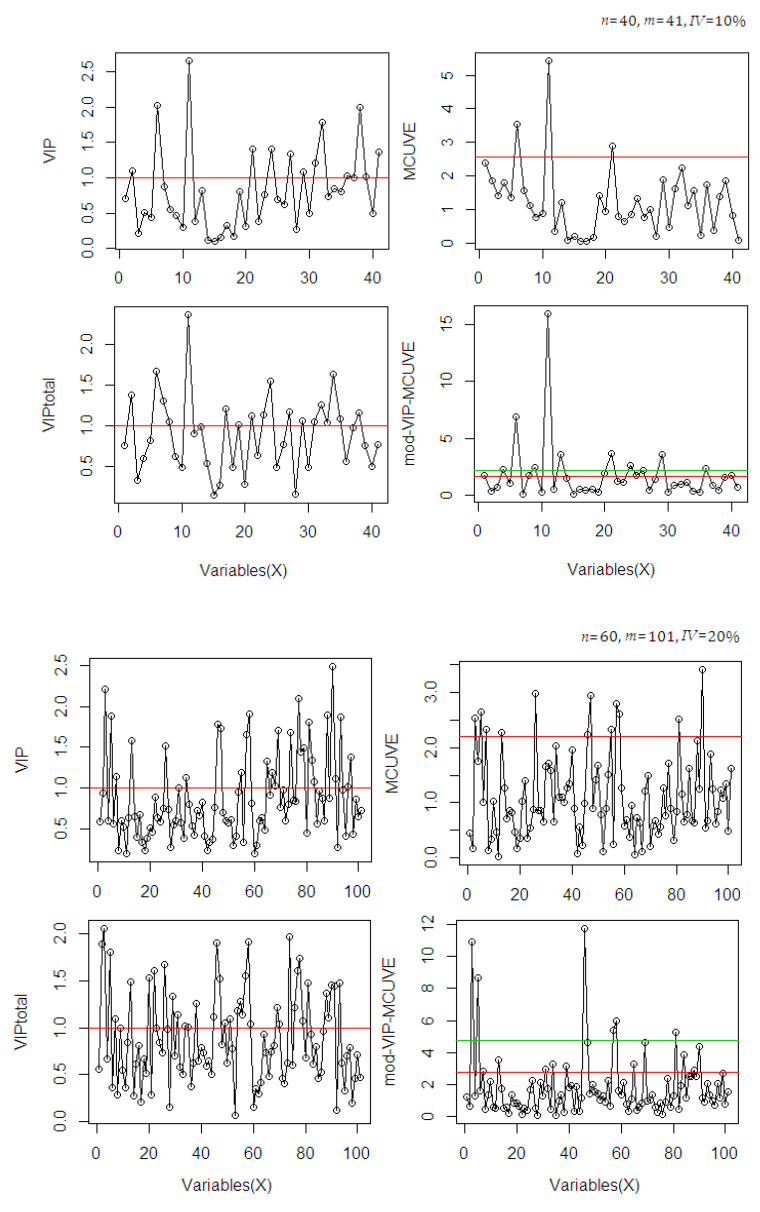

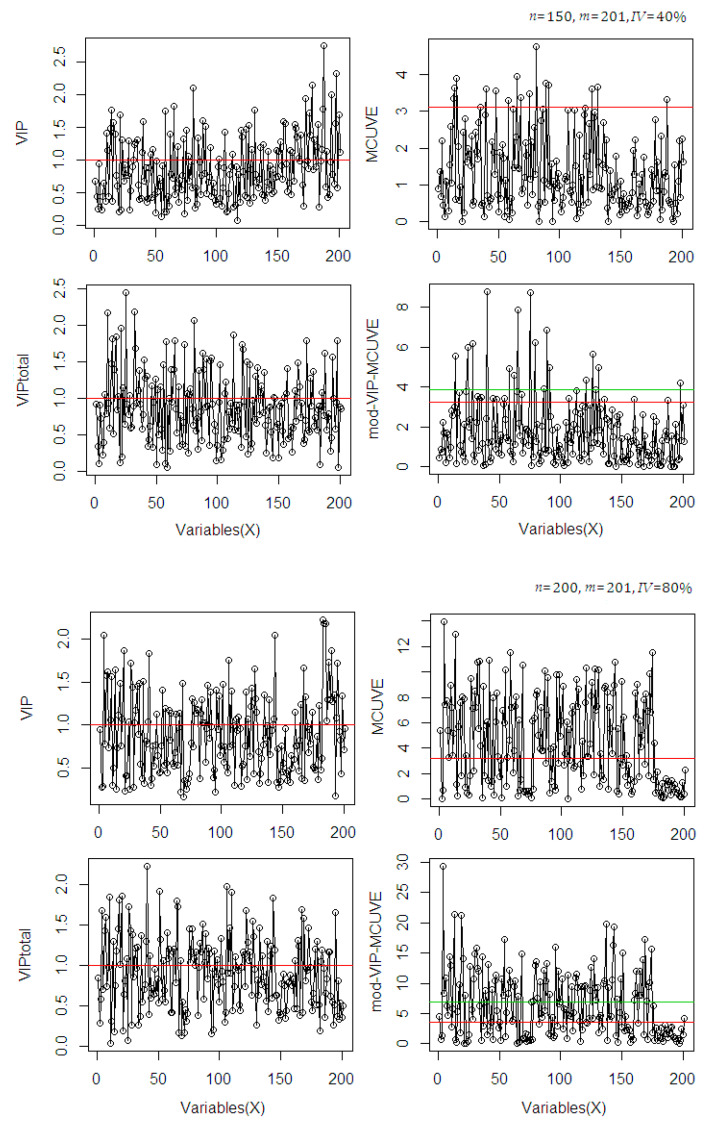
Comparison of the selected relevant variables based on the cut-off criteria in variable selection methods using different dataset scenarios.

The most relevant variables selected by the methods are classified based on their cut-off threshold criterion on the score values. To evaluate the interpretability, the calculations are plotted in Figure 2.

In Figure 2, the selection of variables in each method uses different cut-off criteria. The selection includes the use of the VIP total score because this procedure is also included in the proposed mod-VIP-MCUVE method. For selection, the classical VIP and the VIP total method use VIP score >1, while the MCUVE and the mod-VIP-MCUVE use cut-off threshold criterion. The VIP-total suggests a greater number of relevant variables than the classical VIP. The MCUVE uses standard cut-off threshold criterion then classifies a greater number of relevant variables than the classical VIP and the VIP-total. The proposed mod-VIP-MCUVE uses robust cut-off threshold (red line threshold) then collects a higher number of relevant variables than the MCUVE (green line threshold). The mod-VIP-MCUVE succeeds to downgrade (close to 0) the irrelevant variables and highlight the pertinent variables of the computational process. Using the proposed mod-VIP-MCUVE, the final subset of selected relevant variables guarantees the best prediction capabilities with better accuracy than the other methods.

**Figure 3:**
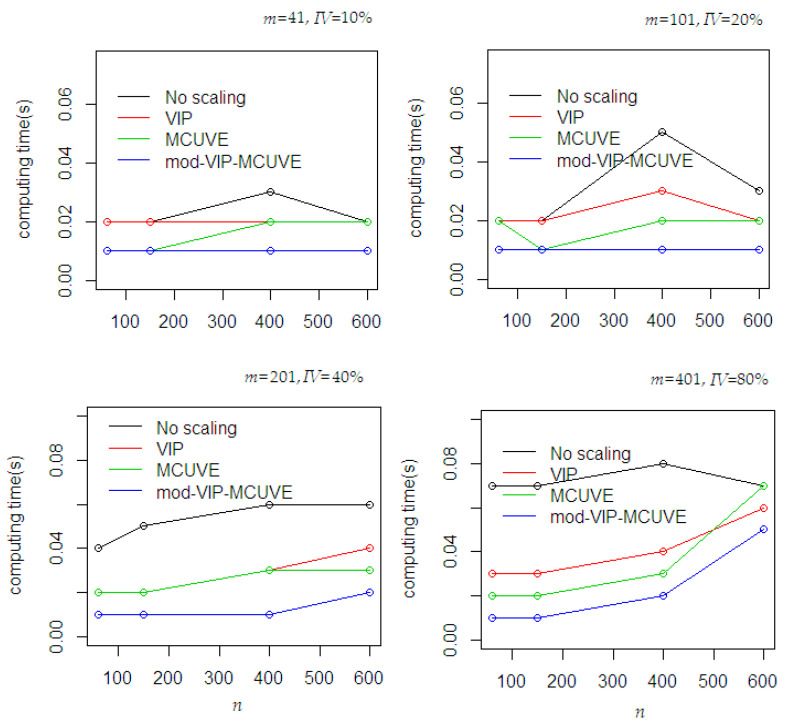
Time-consuming performances between methods during the fitting process using different dataset scenarios (*n =* number of samples, *m* = number of predictors, *IV* = number of important variables).

The computing time performance during the fitting process was recorded to evaluate the efficiency of the proposed mod-VIP-MCUVE method. In Figure 3, the proposed mod-VIP-MCUVE method outperformed the others. Using different sample sizes and numbers of predictor use, the proposed method is still consistent in expediting the convergence speed. The PLSR has the worst performance due to its inefficient computing time even the auto-scaling is naturally applied using its mean and standard deviation in the procedure.

## 5. NIR Spectral Dataset

**Figure 4:**
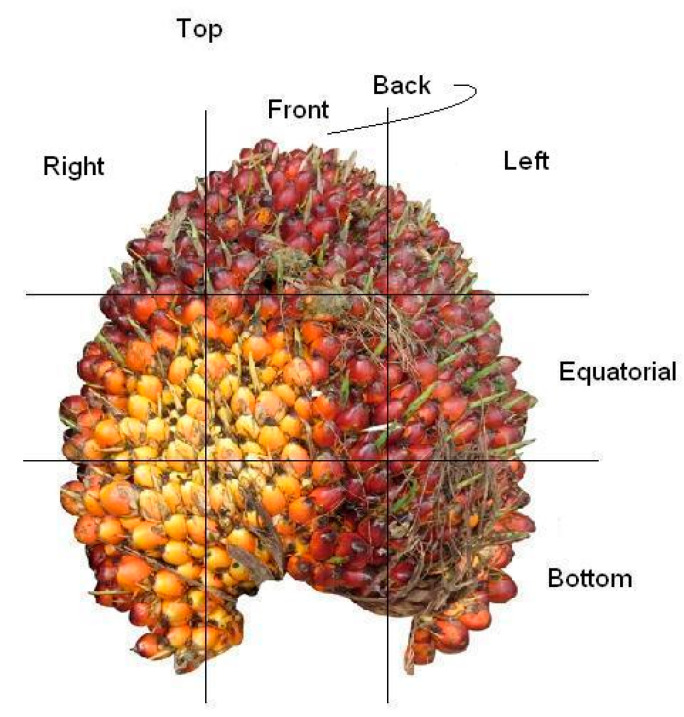
Twelve sampling positions for fruit mesocarp samples of an oil palm fresh fruit bunch.

**Figure 5:**
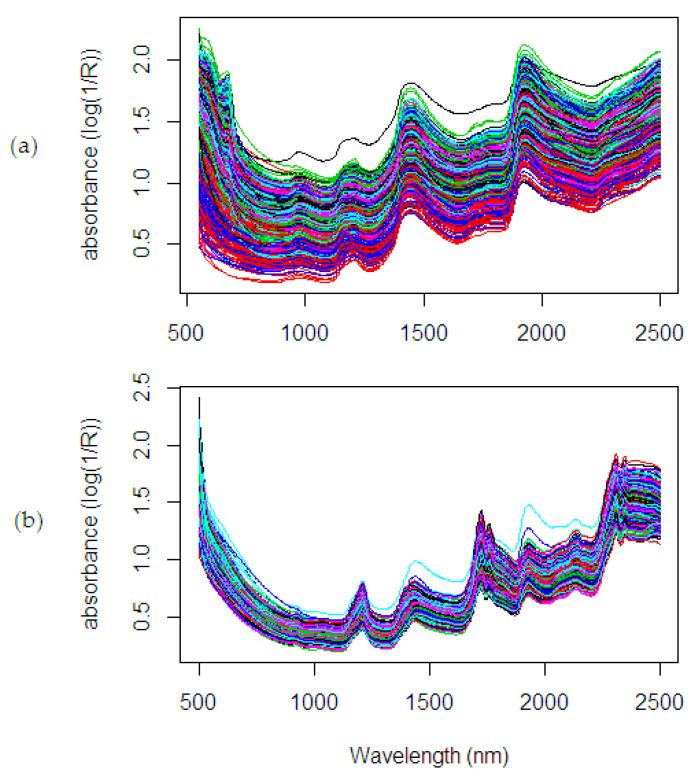
NIR spectra on oil palm fruit mesocarp: (**a**) fresh mesocarp, (**b**) dried ground mesocarp.

A total of 80 fruit bunches were collected from the site of breeding trial in Palapa Estate, PT. Ivomas Tunggal, Riau Province, Indonesia. The source of variability such as planting material (Dami Mas, Clone, Benin, Cameroon, Angola, Colombia), planting year (2010–2012) and ripeness level (unripe, under ripe, ripe, over ripe) were considered for covering as much as possible of the whole range of potential variation in the palm population. Right after harvest, the bunch samples were sent immediately to the laboratory for spectral measurement and wet chemistry analysis. The fruit mesocarp samples were collected from 12 sampling positions by considering the vertical and horizontal lines in a bunch (see Figure 4): bottom-front, bottom-left, bottom-back, bottom-right, equator-front, equator-left, equator-back, equator-right, top-front, top-left, top-back, and top-right. The spectral measurement was done by scanning (in contact) the oil palm fruit mesocarp using a Portable Handheld NIR spectrometer, QualitySpec Trek, from Analytical Spectral Devices (ASD Inc., Boulder, CO, USA). A dataset of NIR spectral data is shown in Figure 5 then was used in evaluating the proposed method. The spectral data as a result of the light absorbance in each j wavelength bands were adopted from Beer–Lambert Law [38] and presented in m×1 column vector $x_{j}$ using the log base 10.

**Figure 6:**
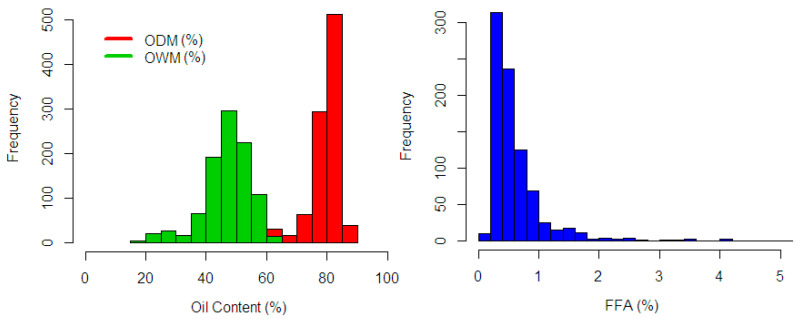
Frequency distribution on response variable: %ODM (red), %OWM (green), and %FFA blue).

The spectra collection was measured three times in each fruit mesocarp sample. The averaged spectra were used in the computation (see Figure 5). There are two sample conditions with different parameters observed in this study: fresh fruit mesocarp and dried ground mesocarp. The fresh fruit mesocarp is used to estimate the percentage of Oil to Dry Mesocarp (%ODM) and Oil to Wet Mesocarp (%OWM), while the dried ground mesocarp is used to estimate the percentage of Fat Fatty Acids (%FFA). These parameters were analyzed through wet chemistry analysis using the standard test methods from the Palm Oil Research Institute of Malaysia (PORIM) [39,40]. The %ODM is calculated in dry matter basis, which removes the weight of water content, while the %OWM uses wet matter basis. As seen in Figure 6, the distribution of the %ODM is 56.38–86.9%, the %OWM is 19.75–64.81%, and the %FFA is 0.17–6.3%. These wide ranges of the distribution showed the possible actual range variation covered in the analysis.

### 5.1. Oil to Dry Mesocarp

In the spectral measurement on fresh fruit mesocarp sample (Figure 5a), each spectrum is composed of 489 wavelengths as data points (range 550–2500 nm: 4 nm interval) with a total spectrum collecting about 960 observations. Here, the importance of the wavelengths is generally unknown, and it needs to be investigated. The selection of the most informative wavelengths in the NIR spectral related to the %ODM in the fresh fruit mesocarp is crucial for further data interpretation. The summary of the fitting performance on the dataset using the calibration model with wavelength selection methods is presented in Table 2.

**Figure 7:**
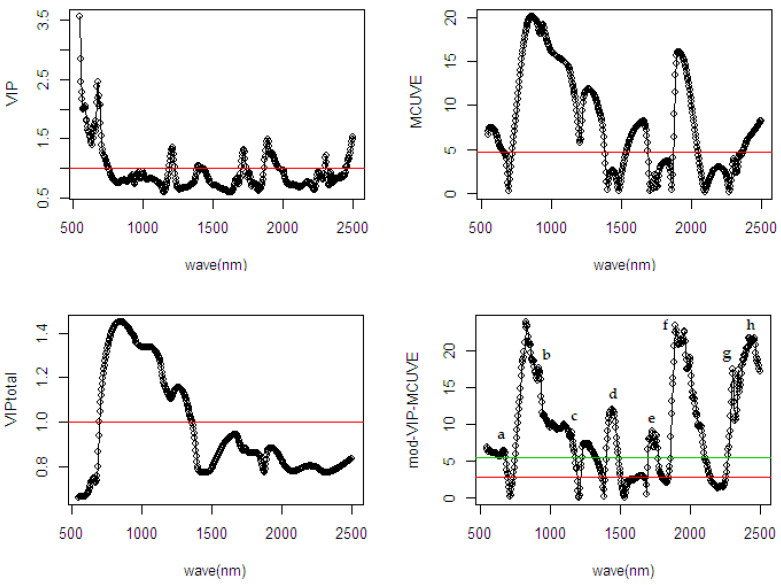
Comparison of selected wavelengths from different wavelength selection methods using spectral data of fresh fruit mesocarp on the %ODM.

As seen in Table 2, the proposed mod-VIP-MCUVE is superior to the other methods. Using the auto-scaling method, the classical PLSR shows the worst performance compared to the other methods with wavelength selection and input scaling applied. The proposed mod-VIP-MCUVE and MCUVE use fewer PLS components than the classical PLSR and VIP method in the fitting process. The classical PLSR suffers overfitting due to the higher number of PLS components used in the model. The VIP method has low accuracy since there are many variables removed in the computation (see Figure 7). Unlike in the MCUVE method, the proposed mod-VIP-MCUVE has better performance and more efficient computationally because of a lower number of PLS components (26 PLS) in the model. The proposed method succeeds in highlighting the most relevant wavelengths and downgrades the influence of irrelevant wavelengths. This result confirms the usefulness of the wavelengths selection and input scaling applied in the input variables which leads to faster convergence speed.

In Figure 7, information regarding the relevant wavelengths related to the %ODM is presented. All the variable selection methods selected the same spectral region, which has the most relevant contribution to the response variable. The methods show a different cut-off threshold to remove the irrelevant wavelengths in the regions. It can be observed that the VIP, MCUVE, and VIP-total removed many irrelevant wavelengths in the model. As assumed earlier, the more wavelengths are excluded in the model the lower the accuracy in the prediction result. In the fourth plot of mod-VIP-MCUVE, the green line shows the old cut-off threshold using previous MCUVE (threshold = 5.486), while the red line is the new proposed robust cut-off threshold (threshold = 2.916). The proposed mod-VIP-MCUVE method shows better cut-off threshold since there is only a smaller number of wavelengths indicated as the most irrelevant wavelengths.

The diffuse selected reflectance [38] is important to identify the relevant wavelengths related to the %ODM. This exhibits their fundamental attribute to the overtone or combination bands involving the molecular stretching and bending absorption over a wide spectral range. The main absorption in the NIR spectral range is produced by the combination and overtone of C-H, O-H, N-H, and C=O groups. The relevant wavelength ranges are indicated through lowercase alphabet notation in the graphic. Based on Figure 7, it is feasible to observe that the well-defined absorption bands are from visible red color (a: 668–684 nm), CH_2_ of oil and O-H of water (b: 936–961 nm), C-H absorption by stretching-bending (c: 1232–1344 nm), first overtone (d: 1404–1444) of C-H stretch O-H of water and C-H of oil and its combinations (e: 1700–1776) of C-O oil and C-H stretching by first overtone, C=O absorption by stretching-bending (f: 1888–2008 nm), C-H second overtone of protein and oil (g: 2296–2360 nm), and corresponding to absorption which associated with the second overtone in C-H of oil (h: 2364–2496 nm).

### 5.2. Oil to Wet Mesocarp

Using a similar set of NIR spectral data from fresh mesocarp as in Section 5.1, the %OWM is used as a response variable. As indicated in Figure 6, the variability of the water content has impacted the shifting of distribution in the response variable. The summary of the fitting performance on the prediction results using different variable selection treatment is presented in Table 3. The comparison shows that the proposed mod-VIP-MCUVE could achieve superior performance than the other methods. It offers the accuracy improvement of RMSE and R^2^ to the VIP, MCUVE, and PLSR methods. Moreover, the number of selected optimal PLS components used in the calibrated model of the proposed mod-VIP-MCUVE is the smallest (17 PLS) which indicates that important information could still be attained even by using fewer variables. This also has proven the necessity to accomplish the wavelength selection before fitting the calibration model.

**Figure 8:**
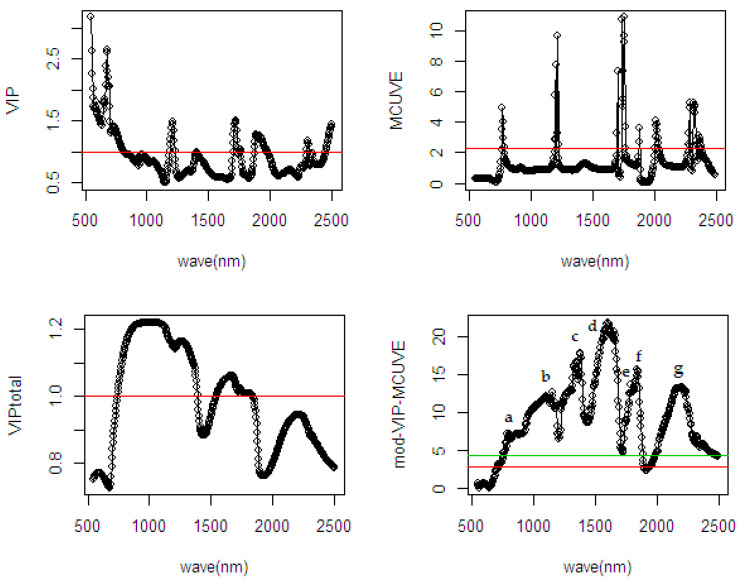
Comparison of selected wavelengths from different wavelength selection methods using spectral data of fresh fruit mesocarp on the %OWM.

In Figure 8, the cut-off threshold in VIP and MCUVE has minimized many wavelengths which forces much important information to become lost. The proposed mod-VIP-MCUVE and new proposed cut-off (red line) succeeds to minimize only the most irrelevant variable and to retain rich information in the spectra. Using old cut-off (green line = 2.881), it has minimized a greater number of wavelengths than the new cut-off (red line = 4.270). From the highlighted wavelength information (mod-VIP-MCUVE) in Figure 8, it is possible to identify the essential molecules in raw NIR spectral and its chemical association to the parameter %OWM. The most relevant wavelengths are O-H of water and CH_2_ of oil (a: 890–950 nm), C-H second overtone and C=O stretch fourth overtone (b: 1140–1166 nm), first and second overtone of the C-H stretching mode (c: 1326–1370 nm), N-H stretch/C-H stretch first overtone (d: 1558–1650 nm), C-H stretch O-H of water and CH of oil and its combinations (e: 1782–1802 nm), O-H stretch/C-O stretch second overtone combination (f: 1830–1854 nm), and C-H stretch/C=O stretch combination of protein and oil (g: 2126–2184 nm).

### 5.3. Fat Fatty Acids

Another NIR spectral dataset (Figure 5b) using a total of 839 observations and 500 wavelengths (in the range 500–2500 nm: 4 nm interval) were collected from dried ground mesocarp sample. The importance of the wavelengths related to the %FFA is unknown. The summary of the fitting performance on the dataset using the calibration model with wavelength selection methods is presented in Table 4.

**Figure 9:**
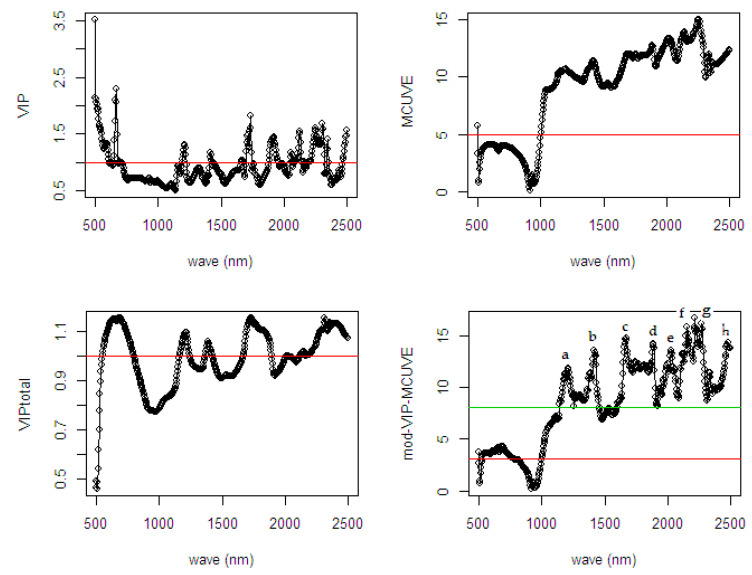
Comparison of selected wavelengths from different wavelength selection methods using spectral data of ground dried mesocarp on the %FFA.

Similar to previous results, the proposed mod-VIP-MCUVE is superior to the classical VIP and the classical PLSR. The performance of the MCUVE is comparable to the mod-VIP-MCUVE. However, the proposed mod-VIP-MCUVE uses less PLS components (25 PLS) than MCUVE, the classical PLSR, and the VIP method in the fitting process. These have proven its efficiency in the computation. The classical PLSR is still suffering overfitting due to a higher number of PLS components used in the model. The VIP method also has low accuracy since there are many variables removed in the computation (see Figure 9). The MCUVE shows its performance is better than the classical PLSR and VIP; however, it is a non-robust method. Again, the proposed mod-VIP-MCUVE method has succeeded in highlighting the most relevant wavelengths and downgraded the influence of irrelevant wavelengths. These results confirmed the usefulness of the wavelengths selection method in the input scaling strategy to improve the classical PLSR model.

As shown in Figure 9, the cut-off threshold in the mod-VIP-MCUVE and MCUVE has succeeded in removing only the most irrelevant wavelengths and keeping the remaining of the relevant variables in the model. The VIP and the VIP-total has removed many variables in the model, and this yields the less relevant variables also included in the removal process. Hence, the less important variables were reduced in the fitting process. As shown in the fourth plot of the mod-VIP-MCUVE method, the old cut-off threshold using previous MCUVE represented as the green line is 8.071, while the new proposed cut-off threshold represented as the red line is 3.039. Based on these thresholds, the number of the irrelevant variables indicated in the mod-VIP-MCUVE is still less than the MCUVE.

The fundamental attribute of diffuse selected reflectance related to the %FFA is crucial to be investigated. The mod-VIP-MCUVE method is used to interpret the selected wavelength, which has well-defined absorption both in the visible and NIR regions. In Figure 9, the most relevant wavelengths are C=O stretch fourth overtone and C-H second overtone (a: 1176–1220 nm), O-H first overtone and C-H of oil (b: 1408–1436 nm), absorption by stretching and bending of C-H stretch first overtone (c: 1652–1688 nm), O-H stretch/C-O second overtone and C=O stretch second overtone (d: 1820–1896 nm), N-H second overtone of protein, absorption by stretching-bending N-H/Amide and C=O stretch second overtone (e: 1996–2040 nm), O-H bend/C-O stretch and C-O-O stretch third overtone, C-H stretch/C=O oil (f: 2116–2288 nm),C-H stretch and C-O of oil (g: 2324–2344 nm), and corresponding to absorption which associated with the second overtone in C-H and C-N-C stretch first overtone of protein (h: 2456–2500 nm).

## 6. Conclusions

In summary, the NIR spectral region contains rich and abundant information that warrants further interpretation using advanced chemometric techniques. The study has shown that the wavelength selection using input scaling method strategy to be promising, particularly with the application on a high dimension dataset such as the NIRS spectral data. The proposed method is robust since it uses robust reliability weight procedure to rescale the original input matrix. According to the evaluation indices, the proposed mod-VIP-MCUVE method confirmed its superiority to the classical PLSR, the VIP method, and the MCUVE method. Using the modified robust cut-off threshold, the proposed method succeeds to highlight the irrelevant wavelengths in the model. Moreover, the proposed method also has the benefit to reduce the data dimension and to improve the model accuracy and computational complexity. The proposed method has even investigated successfully the fundamental attribute of diffuse selected reflectance of the NIRS spectral absorption. This is essential in the improvement of the chemical interpretability. Further, the input scaling procedure using robust selection procedure on optimum number of PLS component is expected to get better improvement in the computational complexity.

## Figures and Tables

**Table 1:** The RMSE, R^2^, RPD, and SE in variable selection methods using Monte Carlo Simulation with different dataset scenarios.

*n*	*m*	*IV*	Method	*n*PLS	RMSE	R^2^	RPD	SE
40	41	10%	PLSR	5	2.130	0.994	12.824	2.164
			VIP	4	2.524	0.991	10.488	2.567
			MCUVE	2	2.016	0.994	13.133	2.050
			mod-VIP-MCUVE	2	2.025	0.994	13.072	2.060
40	101	10%	PLSR	4	9.715	0.940	4.130	9.839
			VIP	5	9.368	0.944	4.283	9.487
			MCUVE	4	10.705	0.927	3.748	10.841
			mod-VIP-MCUVE	4	4.302	0.988	9.327	4.356
40	201	10%	PLSR	N/A	N/A	N/A	N/A	N/A
			VIP	N/A	N/A	N/A	N/A	N/A
			MCUVE	2	15.843	0.834	2.797	16.044
			mod-VIP-MCUVE	2	6.363	0.970	8.353	6.444
60	41	20%	PLSR	8	4.448	0.974	7.005	4.486
			VIP	6	4.848	0.973	6.322	4.889
			MCUVE	5	5.266	0.967	5.812	5.311
			mod-VIP-MCUVE	5	4.443	0.976	7.873	4.447
60	101	20%	PLSR	5	10.248	0.968	5.601	10.335
			VIP	4	16.726	0.914	3.432	16.867
			MCUVE	7	13.579	0.943	4.227	13.693
			mod-VIP-MCUVE	4	5.143	0.992	11.160	5.187
60	201	20%	PLSR	N/A	N/A	N/A	N/A	N/A
			VIP	N/A	N/A	N/A	N/A	N/A
			MCUVE	2	33.192	0.735	2.544	33.472
			mod-VIP-MCUVE	3	10.678	0.962	11.028	10.768
150	41	40%	PLSR	6	6.359	0.971	5.931	6.381
			VIP	7	7.183	0.963	5.242	7.207
			MCUVE	5	7.097	0.964	5.306	7.121
			mod-VIP-MCUVE	4	6.291	0.971	5.933	6.297
150	101	40%	PLSR	9	8.949	0.979	7.379	8.979
			VIP	8	12.947	0.958	5.047	12.991
			MCUVE	6	11.023	0.970	5.850	11.060
			mod-VIP-MCUVE	5	8.858	0.987	7.761	8.861
150	201	40%	PLSR	5	14.798	0.975	6.406	14.847
			VIP	5	27.199	0.917	3.485	27.290
			MCUVE	5	23.706	0.937	3.999	23.785
			mod-VIP-MCUVE	2	14.809	0.975	5.347	14.852
400	41	60%	PLSR	5	9.424	0.967	5.515	9.436
			VIP	9	10.057	0.962	5.157	10.070
			MCUVE	7	9.991	0.963	5.200	10.003
			mod-VIP-MCUVE	7	9.423	0.969	5.549	9.435
400	101	60%	PLSR	6	14.258	0.972	6.025	14.275
			VIP	9	16.240	0.964	5.290	16.260
			MCUVE	7	15.424	0.968	5.571	15.443
			mod-VIP-MCUVE	7	14.310	0.968	5.611	14.329
400	201	60%	PLSR	10	14.258	0.972	6.025	14.275
			VIP	14	16.240	0.964	5.290	16.260
			MCUVE	10	15.424	0.968	5.571	15.443
			mod-VIP-MCUVE	7	14.310	0.968	5.611	15.319
600	41	80%	PLSR	10	10.789	0.966	5.469	10.798
			VIP	8	11.072	0.965	5.328	11.081
			MCUVE	8	11.139	0.964	5.297	11.147
			mod-VIP-MCUVE	8	10.985	0.967	5.471	10.994
600	101	80%	PLSR	12	16.264	0.969	5.737	16.278
			VIP	10	16.998	0.967	5.495	17.012
			MCUVE	9	17.019	0.967	5.483	17.033
			mod-VIP-MCUVE	9	16.210	0.970	5.781	16.223
600	201	80%	PLSR	12	20.988	0.975	6.305	21.005
			VIP	12	22.276	0.971	5.942	22.295
			MCUVE	8	22.588	0.971	5.855	22.607
			mod-VIP-MCUVE	8	20.295	0.976	5.940	20.314

Note: *n*PLS is a number of optimum PLS components used in the PLSR model.

**Table 2:** The RMSE, R^2^, RPD, and SE in variable selection methods using %ODM data.

Dataset	Methods	*n*PLS	RMSE	R^2^	RPD	SE
%ODM	PLSR	29	3.267	0.652	1.607	3.269
	VIP	29	3.011	0.657	1.702	3.013
	MCUVE	26	3.107	0.633	1.650	3.108
	mod-VIP-MCUVE	26	3.009	0.659	1.725	3.011

Note: *n*PLS is a number of optimum PLS components used in the PLSR model.

**Table 3:** The RMSE, R^2^, RPD, and SE in variable selection methods using %OWM data.

Dataset	Methods	*n*PLS	RMSE	R^2^	RPD	SE
%OWM	PLSR	20	4.558	0.651	1.693	−0.067
	VIP	20	4.506	0.659	1.713	0.062
	MCUVE	18	4.461	0.666	1.730	−0.016
	mod-VIP-MCUVE	17	4.400	0.675	1.754	0.060

Note: *n*PLS is a number of optimum PLS components used in the PLSR model.

**Table 4:** The RMSE, R^2^, RPD, and SE in variable selection methods using %FFA data.

Dataset	Methods	*n*PLS	RMSE	R^2^	RPD	SE
%FFA	PLSR	28	0.270	0.730	1.924	0.271
	VIP	28	0.266	0.734	1.932	0.267
	MCUVE	26	0.264	0.736	1.946	0.265
	mod-VIP-MCUVE	25	0.265	0.735	1.940	0.266

Note: *n*PLS is a number of optimum PLS components used in the PLSR model.
